# Habitat Fragmentation and Species Extirpation in Freshwater Ecosystems; Causes of Range Decline of the Indus River Dolphin (*Platanista gangetica minor*)

**DOI:** 10.1371/journal.pone.0101657

**Published:** 2014-07-16

**Authors:** Gill T. Braulik, Masood Arshad, Uzma Noureen, Simon P. Northridge

**Affiliations:** 1 Sea Mammal Research Unit, Scottish Oceans Institute, University of St. Andrews, St. Andrews, Fife, United Kingdom; 2 World Wildlife Fund-Pakistan, Lahore, Pakistan; 3 Wildlife Conservation Society, Zanzibar, United Republic of Tanzania; Natural History Museum of Denmark, Denmark

## Abstract

Habitat fragmentation of freshwater ecosystems is increasing rapidly, however the understanding of extinction debt and species decline in riverine habitat fragments lags behind that in other ecosystems. The mighty rivers that drain the Himalaya - the Ganges, Brahmaputra, Indus, Mekong and Yangtze - are amongst the world’s most biodiverse freshwater ecosystems. Many hundreds of dams have been constructed, are under construction, or are planned on these rivers and large hydrological changes and losses of biodiversity have occurred and are expected to continue. This study examines the causes of range decline of the Indus dolphin, which inhabits one of the world’s most modified rivers, to demonstrate how we may expect other vertebrate populations to respond as planned dams and water developments come into operation. The historical range of the Indus dolphin has been fragmented into 17 river sections by diversion dams; dolphin sighting and interview surveys show that river dolphins have been extirpated from ten river sections, they persist in 6, and are of unknown status in one section. Seven potential factors influencing the temporal and spatial pattern of decline were considered in three regression model sets. Low dry-season river discharge, due to water abstraction at irrigation barrages, was the principal factor that explained the dolphin’s range decline, influencing 1) the spatial pattern of persistence, 2) the temporal pattern of subpopulation extirpation, and 3) the speed of extirpation after habitat fragmentation. Dolphins were more likely to persist in the core of the former range because water diversions are concentrated near the range periphery. Habitat fragmentation and degradation of the habitat were inextricably intertwined and in combination caused the catastrophic decline of the Indus dolphin.

## Introduction

Fresh waters are experiencing declines in biodiversity far greater than those in the most affected terrestrial ecosystems [1], [2]. Dam construction has dramatically increased habitat fragmentation and degradation in freshwaters, which is likely to have incurred a large unredeemed extinction debt [3]. However, this debt is not yet well quantified or understood, as metapopulation ecology in freshwaters has lagged behind similar work in other habitats, such as tropical forests [4], [5], [6]. A fundamental, yet unanswered question for conservation biology is how rapidly freshwater species disappear from river fragments and which factors influence the extinction of freshwater species in habitat patches. We investigate this issue using the example of the highly fragmented Indus River system and its endemic, endangered freshwater cetacean.

The great rivers that drain the Himalaya are amongst the world’s most biodiverse freshwater ecosystems, but they are increasingly under threat as the emerging nations of China, India, and Pakistan and the countries of Southeast Asia scramble to harness hydropower and provide water for expanding agrarian economies, in the midst of increasing water scarcity and climatic uncertainty [7], [8], [9]. Many hundreds of new dams and other water development projects are planned or under construction in the region, including mainstem and tributary mega-dams, run-of-the-river hydroelectric schemes, irrigation barrages, and inter-basin water transfers [10], [11]. The Himalayan region will soon have the highest concentration of dams in the world [12]. The combined effects of these activities are predicted to cause rapid and escalating hydrological change and habitat fragmentation that will negatively impact riverine biodiversity and ecosystem services [9], [13].

The freshwater cetaceans that inhabit the largest Himalayan rivers, the Indus, Ganges, Brahmaputra, Yangtze, Mekong, and Ayeyarwady, collectively form one of the world’s most endangered groups of mammals, each listed as endangered or critically endangered on the IUCN RedList [14], and, in the case of the Yangtze River dolphin (*Lipotes vexillifer*), the species has probably been extinct since the mid-2000’s [15]. River dolphins are iconic species that can serve as charismatic flagships for conservation of freshwater ecosystems; but they are poorly understood and increasingly threatened. This is exemplified by the demise of the Yangtze River dolphin which disappeared so quickly that a comprehensive evaluation of the causes of its decline was not conducted until after it was presumed extinct [16].

**Figure 1:**
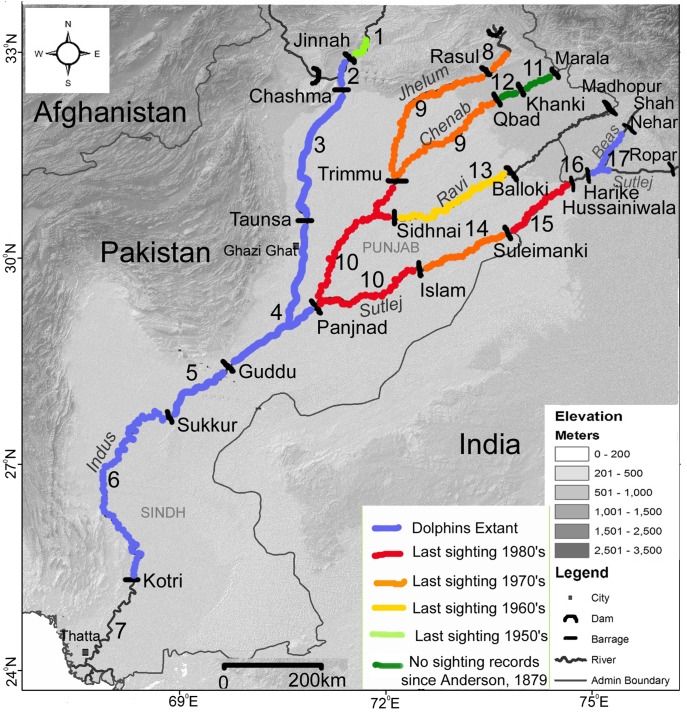
Map of the lower Indus River system. Rivers and barrages are named, and each river section is numbered and coloured according to whether river dolphins are extant, or the approximate date that they were extirpated (see Table A1 for details).

**Figure 2:**
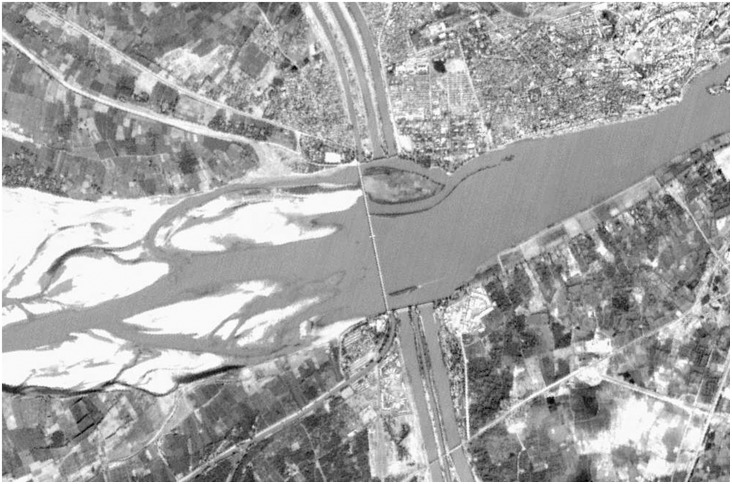
Aerial photograph of Sukkur Barrage. Image shows the seven canals diverting water out of the river, and demonstrates the dramatically reduced flows downstream (river flow direction right to left).

We examine the pattern, and causes of range decline of the Indus dolphin (*Platanista gangetica minor*), an obligate freshwater cetacean endemic to the Indus River system, which, after more than 150 years of barrage (gated-dam) construction and removal of water to feed irrigated agriculture, is one of the most fragmented and modified rivers in the world. The distribution of the Indus dolphin was carefully documented in the 1870’s, (just prior to the first major barrage being constructed) and at that time the dolphin inhabited the entire lower Indus River system from the delta with the Indian Ocean, to the foothills of the Himalayas in what is now India and Pakistan [17]. By the early 1990’s, Indus dolphins had undergone an 80% reduction in range, having been extirpated from the upper and lower reaches of the Indus and four of the largest tributaries [18]. They are now confined to five contiguous ‘river sections’ on the Indus mainstem in Pakistan, separated by barrages, and in the Beas River, in India (Fig. 1) [19], [20]. Details of when dolphins were extirpated from different parts of their former range are vague, and the causes not clearly understood. However, the construction of twenty irrigation barrages between 1886 and 1971 (gated-dams used for water diversion) that fragmented the dolphins historical range into 17 river sections (numbered 1–17 on Fig. 1), and large-scale water abstraction for irrigation rendering many sections of river almost dry for many months, certainly played a role (see Fig. 2) [19], [21].

In this paper we document the spatial and temporal dynamics of the Indus River dolphin range decline, and then use a series of regression models to determine the causes of the spatial pattern of decline, the timing of subpopulation extirpation, and the speed of subpopulation disappearance after habitat fragmentation. Greater understanding of how the Indus dolphin has responded to the presence of dams and water diversions within its habitat demonstrates how we may expect other vertebrate populations to respond as planned dams and water developments come into operation elsewhere. The results provide important and relevant insights into factors influencing species extinction in fragmented riverscapes.

## Methods

### Last Dolphin Sighting Date

The status of each of the six extant dolphin subpopulations is fairly well understood [19], [22], but there is little information on when or why dolphins disappeared from the 11 river sections where dolphins are presumed extirpated (Fig. 1). In 2007, we compiled historical dolphin sighting dates and locations from areas in which dolphins are believed to have been extirpated, by conducting community interviews, and a review of historical literature. GB received clearance from the Pakistan Home Department for the survey, and as there was no appropriate ethics or review board in Pakistan to provide approval no other permits were required. Depending on location, dolphins are believed to have disappeared between 20 and 80 years ago, so the short structured interviews targeted elderly riverside inhabitants old enough to have seen dolphins in their lifetimes. The objective of the interviews was explained to potential interviewees and they provided their verbal, rather than written, consent to participate as the majority were illiterate. All interviews were anonymous. A calendar of significant local historical events was compiled to assist informants in recalling dates correctly. A last dolphin sighting date (LDSD) was allocated to each river section based on the most recent dolphin sighting that we identified. We did not attempt to identify the exact extinction date of each subpopulation [23], but used the LDSD as a general indicator of when dolphins disappeared [24]. Inexact dates were assigned to 5 year intervals, for example, if the date was early 1970s, a date of 1972 was assigned, if it was mid 1970s, 1975 was designated, and if it was late 1970s the date used was 1978.

### Identifying the Causes of Range Decline

The following seven explanatory variables that may have contributed to the Indus dolphin range decline were determined for each of the 17 sections of the dolphin’s former range:

1. *Fragmentation date -* The year that each river section was created; assigned as the date that the second of the two bounding barrages became operational. For the river section upstream of Harike barrage in India, the isolation date was assigned as the completion date of Hussainiwala barrage which is located only 30 km downstream of Harike.
2. *River length* - The number of river kilometres between two barrages.
3. *Proximity to range edge* - The distance along the river’s course from the former dolphin distributional limit recorded by Anderson [17], to the barrage located closest to the range core.
4. *Size of river* - The mean annual discharge in Million Acre Feet reported for each river prior to implementation of the Indus Water Treaty in 1960 [25]. This illustrates original river size prior to large-scale water abstraction.
5. *Confluences* - The number of river confluences within each river section was included as an indicator of favourable habitat, as Indus dolphins occur with higher frequency at confluences [26].
6. *River slope* - The slope within each river section was calculated as the drop in elevation between the up and downstream barrages, or upstream range limit in the case of peripheral segments, divided by the length of river. Slope exerts a direct effect on flow velocity and sediment transport and therefore may influence dolphin habitat.
7. *Dry season river discharge* - River discharge data were obtained for all twelve barrages and two dams north of Guddu on the Indus River system in Pakistan for the period July 2008 to April 2013 (4 years 9 months). The daily discharge below Guddu and Kotri barrages was obtained from October 2010 to April 2013 (∼2 ½ years), and discharge below Sukkur barrage was obtained from April 1994 to January 2000, and October 2010 to April 2013 (8 years 3 months). Occasional missing data were interpolated. It is very low flows that are likely to adversely impact dolphins, therefore median daily discharge during the dry season (1^st^ October to 31^st^ March) was determined using the years for which there was an entire dry season’s data. Mean monthly discharge was available above and below Harike and Hussainiwala barrages in India from January 2009 to December 2011, and for these two river sections the median of the mean monthly dry season discharge was used. The number of years of data available differed according to barrage but the temporal discharge pattern was predictable and similar across years in each location.

Generalised Linear Models (GLMs) and a survival analysis were used with the seven explanatory variables described above as predictors of the continued presence of river dolphins in each of the 17 river sections. Generalised Additive Models were used in the initial data exploration to visually investigate whether the relationship between the predictor and explanatory variables was linear, and, which type of transformation could be used to best account for non-linearity. Three sets of models were developed, with objectives summarised below:

#### Spatial pattern of dolphin persistence

The objective of the first set of models was to identify which factors best explained the observed geographic pattern of range decline. The presence or absence of dolphins in each river section was modelled using a GLM and a binomial error distribution, with presently extant populations coded as 1 and extirpated populations coded as 0. The best fitting models were then used to predict the probability that dolphins are still present in the Harike-Hussainiwala river section on the India-Pakistan border (Fig. 1: no. 16), where dolphin presence is unknown.

#### Temporal pattern of decline

The second model set included only sections where dolphins have been extirpated and examined which factors influenced when dolphins disappeared. The number of years since dolphins were sighted (as of 2013) was the response variable modelled using a GLM with a quasi-Poisson error distribution.

#### Time to extirpation

The third model set used a survival function to investigate which factors influenced the speed with which dolphin populations were extirpated following their isolation between barrages. We used the Kaplan–Meier estimate of survival which allows for the inclusions of censored data, in this instance allowing for the inclusion of river sections where dolphins have not yet been extirpated, as well as those where dolphins have disappeared. Each river section was qualified with a status assignment, where 1 = dolphins extirpated, and 0 = dolphins extant [27]. The time to extirpation was calculated as the number of years between the fragmentation date and either the LDSD if dolphins have been extirpated, or the current year, 2013, where they are still present. Time to extirpation and status together were the predictor variable (the Kaplan-Meier survivorship object) modelled using the ‘survreg’ function in the survival library of the program R [28]. Both an exponential and a Weibull error distribution were tested, and the Weibull distribution was selected as it provided a significantly better fit to the data (ΔAIC 15.17) [27].

All models were implemented using the program R 2.15.1 [28]. Logit, probit and cloglog link functions were included in global models and the logit function, which resulted in the best fit, applied. Variance Inflation Factors (VIFs) that demonstrate the degree of collinearity between variables were generated from the maximal models and collinear variables removed until VIF scores were less than five [27]. Three two-way interactions that described potentially meaningful relationships between variables (fragmentation date and dry season discharge, fragmentation date and river length, and river length and proximity to range edge) were included, as well as second and third order polynomials of significant variables. The binomial and survival models were simplified using backwards stepwise selection based on Akaike’s Information Criteria (AIC). Quasi-Poisson models were selected on the basis of quasi-AIC (QAIC) scores, and non-significant terms were sequentially dropped based on their levels of significance. Models separated by at least two AIC/QAIC points were assumed to be significantly different [29]. Goodness of fit for the GLMs was measured by determining the proportion of the total deviance explained by the final model. Model plots were examined for non-normality of errors, statistical independence of observations, heteroscedasticity and influential points [30].

## Results

### Pattern of Range Decline

Historical dolphin sightings were obtained for all river sections formerly occupied by dolphins except for the area downstream of Kotri Barrage to the delta (Fig. 1: no. 7) and the stretch from Harike to Hussainiwala barrage (Fig. 1: no. 16) which is close to the India-Pakistan border. Our focus on retired fishermen and on areas from which dolphins have already been extirpated meant that the communities were forth-coming with information because it was not regarded by them as sensitive. However, there were very few elderly members of each fishing community and our pool of available informants was consequently small (n = 57). 79% of informants were, or had been, full-time commercial fishermen or contractors and the remainder were part-time subsistence fishermen. There was no significant difference in the age of informants interviewed at each barrage (GLM p = 0.7) but older individuals were significantly more likely to have seen dolphins than younger informants (GLM p<0.01). We found no evidence that dolphins persist in any of the Indus tributaries in Pakistan. Of 17 sections of river, dolphins are extant in six, have been extirpated from ten, and in one border area that could not be surveyed (no. 16), dolphin presence or absence is unknown (Fig. 1).

### Causes of Range Decline

For each river section, dolphin presence or absence, estimated LDSD, and the time to extirpation were compiled along with the physical characteristics and these data included in each of the models (Table S1). On the upper Chenab River (Fig1: no 11 & 12) the LDSD is that reported by Anderson in 1879. Time to extirpation was not calculated for these two sections reflecting the lack of recent sighting evidence and uncertainty of the extirpation date.

Where dolphins are still extant the median monthly dry season river discharge averaged 30,830 cusecs (ranged 7,224–47,040 cusecs), as compared to an average of 8,022 cusecs (range 0–38,000 cusecs) in locations from which dolphins have been extirpated. In general, sections of river where dolphins are still present were fragmented by barrages later, are further from the range periphery, are of longer length, have a shallower slope and greater dry season discharge than river sections where dolphins are no longer found.

**Figure 3:**
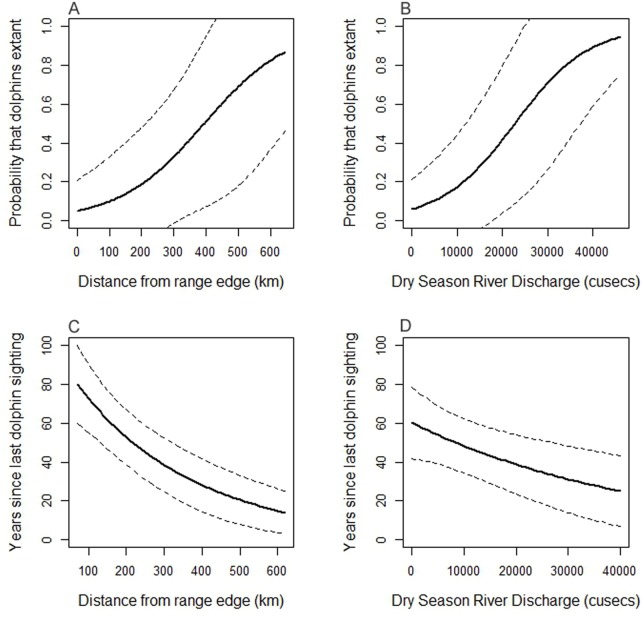
Significant relationships retained in GLM models of the causes of the spatial and temporal pattern of Indus dolphin decline. The figure demonstrates the probability that an Indus dolphin subpopulation is extant according to A) proximity to the edge of the former range and B) median dry season river discharge, and the relationship between the number of years since a dolphin was sighted and C) distance from the historical distributional limit, and D) median dry season river discharge.

Sixteen river sections, 6 where dolphins are extant and ten where they have been extirpated, were included in the spatial GLM models. The VIFs generated from the full model indicated that river discharge and slope were collinear. Slope was considered to be less important than discharge in explaining dolphin range decline because it has not changed substantially in hundreds of years, and it was therefore removed from further candidate models. The final model that best explained the observed spatial pattern in Indus dolphin range decline retained the explanatory variables dry season river discharge and distance from range edge (Table 1). The probability that an Indus dolphin subpopulation is still extant increases with increasing distance from the range edge, and with increasing dry season river discharge (Fig. 3). The final spatial model predicted only a 2.6% probability that dolphins are still extant in the Harike-Hussainiwala river section in India that was not surveyed for dolphins. This is not unexpected given that this section has very low dry season discharge and is near the periphery of the dolphin’s range, both factors that increase the likelihood of subpopulation extirpation. Although the linear arrangement of river segments might suggest a lack of statistical independence, there was no clumping of residuals according to the geographic position of the river segments, and the independence of observations was further shown by the Durbin-Watson test of correlated errors (p = 0.24).

**Table 1:** Summary of spatial range decline model output.

*Model*	*AIC*	*Δ AIC*	*% explained Deviance*	*n*	*Deviance*						
					Q	Range	Is. Date	L	Conf	Size	USD: Is. Date
1	18.26	1.82	32.6	1	6.91	-					
**2**	**16.44**	**-**	**50.7**	**2**	**7.88**	**2.84**	**-**	**-**	**-**	**-**	**-**
3	17.72	1.28	54.1	3	7.88	2.84	0.72	-	-	-	-
4	19.67	3.23	54.3	4	7.88	2.84	0.68	0.08	-	-	-
5	21.64	5.20	54.4	5	7.88	2.84	0.42	0.31	0.07	-	-
6	23.64	7.20	54.5	6	7.88	2.84	0.42	0.31	0.07	-	0.001
7	25.16	8.72	56.7	7	7.88	2.84	0.79	0.31	0.07	0.0001	0.11

n = number of covariates, Is. Date = Isolation Date, L = Length of river section, Range = Distance from range edge, Size = River size, Conf = confluences, Q = River discharge, USD: Is. Date = Interaction between Isolation Date and River Discharge. Model in bold was the final selected model.

The variables that described the temporal pattern of Indus dolphin subpopulation extirpation were the same as those that influenced the spatial pattern of decline: dry season river discharge and distance from former range limit (Table 2). Within areas from which dolphins have disappeared, they were extirpated earlier from river sections where discharge was lower, and from those sections located near the periphery of the subspecies former range (Fig. 3).

**Table 2:** Summary of temporal range decline model outputs.

*Model*	*AIC*	*Δ AIC*	*% explained Deviance*	*n*	*Deviance*						
					Q	Range	Is. Date	L	Conf	Size	USD*Is. Date
1	17.57	1.36	56.75	1	-	115.36	-	-	-	-	-
**2**	**16.21**	**-**	**76.76**	**2**	**40.67**	**115.36**	**-**	**-**	**-**	**-**	**-**
3	17.80	1.59	79.17	3	40.67	115.36	-	-	-	-	4.91
4	17.54	1.33	92.62	4	52.06	115.36	-	-	-	6.66	14.20
5	19.50	3.29	92.85	5	52.06	115.36	-	-	6.60	6.66	8.11
6	21.46	5.25	93.12	6	52.06	115.36	0.75	-	6.60	6.66	7.9
7	23.39	7.18	93.49	7	52.06	115.36	0.60	0.33	9.16	6.66	5.89

n = number of covariates, Is. Date = Isolation Date, L = Length of river section, Range = Distance from range edge, Size = River size, Conf.  = confluences, Q = River discharge. Model in bold was the final selected model.

In river sections where dolphins have been extirpated, the mean time from fragmentation to the LDSD was 50 years (SD = 23, range = 9–76). For river sections where dolphins are still extant, the mean time from subpopulation isolation to present (2013) was 57 years (SD = 15, range = 42–86). Thirteen river sections were included in the survival model (number 2, 10, 12 and 16 were excluded because of missing data) and the slope parameter was included. The final survival model retained four variables: median dry season river discharge, isolation date, length of river section and slope. Dolphin subpopulations were extirpated more quickly in sections with low dry season river discharge. Subpopulations persisted longer where the river slope is more gentle (e.g. in the lower reaches) and those that were isolated between barrages a long time ago persisted for longer than those in more recently subdivided river sections. Fifty years after Indus dolphins were isolated between barrages there is a less than a 50% chance that they will still be extant, and after 100 years this probability drops to 37%.

## Discussion

### Model evaluation

The river discharge data used in these models were from the last ten years but they explained well the pattern of dolphin decline that occurred decades ago. Although river discharge varies from year to year, and has generally declined, the relative discharge among barrages (e.g. the spatial relationship) has remained constant with the same locations consistently reporting high (e.g. the upper Indus) and low discharge (e.g. Indus tributaries) over time. Therefore, the assumption implicit in this analysis that the present spatial pattern of discharge reflects that present during the period leading up to dolphin extirpation is not unreasonable.

As for terrestrial habitats, such as forest fragments, we would have expected to see a relationship between species extinction and habitat patch size [5]. In fact, length of river section was one of the first variables to be excluded in candidate models. This may be because only the current configuration of 17 comparatively small habitat fragments were included in the models, and we did not consider the progression of escalating habitat fragmentation and concomitant diminishing fragment size over time. To investigate this, we constructed an additional model considering all 33 river sections that have existed since the onset of barrage construction (Table A2) in a binomial GLM, with dolphins recorded as extirpated (0) or still extent (1) in each river section at the point it was further subdivided. Explanatory variables were 1) Length of river section, 2) Isolation Date, and 3) End Date, taken as the year a new barrage was completed resulting in the sections further subdivision. When considering the entire history of habitat fragmentation, the models showed that dolphins were significantly more likely to be extirpated in smaller fragments (p<0.05), and that this relationship was independent of fragment creation date or duration. The link between species extirpation and habitat fragment size has been clearly established in terrestrial habitats for several species groups [5] but this is one of the first studies to show a similar relationship in riverscapes. It underlines the great importance of maintaining large sections of intact river habitat to sustain tropical aquatic biodiversity.

River discharge and distance from range periphery provided a good fit to the range decline data, explaining more than 76% of the deviance in the temporal model and 50% in the spatial model. However, three other aspects that may have also have played a role in the dolphins decline were not included as explanatory variables because of a lack of suitable data. These are a) water quality, b) incidental capture in fishing gear and c) hunting. The possible contributions of these to the Indus dolphin decline are discussed below.

The magnitude of surface water pollution in Pakistan has increased at a dramatic rate over the last ten years and more than 90% of industrial and municipal effluents enter water courses untreated [31]. The Indus tributaries flow through the industrial and agricultural heartland of Pakistan and are more polluted than the Indus River itself which has a greater dilution capacity and passes through remoter areas. There has been no systematic monitoring of river water quality that could provide data for this analysis. However, dolphins had already been extirpated from most areas prior to significant declines in water quality which occurred in the 1980s and 90s, and this asynchronous timing indicates that pollution was not primarily responsible for the dolphins’ decline.

Mortality from accidental capture in fishing gear is considered to be the greatest threat to most cetacean populations [32]. In the past, the Indus River main channel was not intensively fished because the water was too swift for easy manoeuvrability of oar-powered boats, and instead fishing focused on side channels and adjacent pools that are rarely used by dolphins [33]. Since 2010 changing fishing practices in Sindh Province have led to an increase in dolphin mortality, however, prior to this there are very few records of incidental capture of dolphins in fishing gear and this is not likely to be a large factor in the decline of the Indus dolphin.

Indus dolphins were killed for food, oil and medicine until the late 1970s when the animal became legally protected [17], [34]. Information on dolphin hunting is sparse and un-quantified and records refer only to hunting on the Indus River, where dolphins are still extant. Although it is possible that dolphins were hunted throughout the river system, there is no evidence that this was so, and the fact that they persist in the places that hunting is reported to have been intense, and have disappeared from places where hunting was not reported, suggests that this was unlikely to have been the cause of the subspecies’ decline. However, the timing of reported hunting coincides with the period of decline and without more information, it is not possible to completely discount the role of hunting.

For the majority of the year the gates on all barrages are lowered to divert water into canals, and the physical opening is sufficiently small that it would be difficult or impossible for dolphins to pass through the gates and between different sections of river. It has been hypothesized that there may be consistent or frequent movements of dolphins through some barrages and between subpopulations [21], [35]. It has also been theorised that due to the high water velocity and turbulence often found within the barrage gates it would be more likely for animals to move down-, rather than up-stream, and that this would lead to the downstream migratory attrition of upstream subpopulations [18], [36]. The only evidence of this was obtained in 2009, when, during the annual canal maintenance period, which is one of the brief periods in the year when no water is diverted and barrage gates are fully open for several weeks, one radio-tagged Indus dolphin was recorded to move through the gates on Sukkur barrage in both an up- and down-stream direction (WWF-Pakistan unpublished). Each barrage is quite different in terms of design, location and operation and one dolphin moving through Sukkur barrage does not prove that this occurs at other barrages, or that it is a regular occurrence. Therefore, for the purposes of this study, and in the absence of evidence to the contrary, we assume no significant migration between dolphin subpopulations. It is possible that future research may demonstrate that dolphins do move across some, or all, barrages with regularity and if the movement of individuals is primarily in one direction this would be another important factor to consider in the extinction dynamics. The pattern of subpopulation persistence near the range core, and earlier extirpation of subpopulations near the range periphery could perhaps be partially explained by the consistent downstream migration of animals, but again this pattern is disrupted by the presence of dolphins in the Beas River.

### Causes of Indus dolphin range decline

The clear result of this study was the relationship between low dry season river discharge and the decline of the Indus dolphin. Reduced flows directly impact dolphins by reducing the physical space available to them, reducing average water velocity and depth and increasing water temperatures. Flow regulation is also likely to indirectly impact river dolphins due to declines in fish diversity, the dominance of generalist fish species, and increased success of invasive species [37], [38]. The dampened flood peaks typically associated with dams and diversions reduce the frequency, extent and duration of floodplain inundation that determines how long fish can gain access to nursery habitat and food. Water abstraction also exacerbates and concentrates existing anthropogenic threats, for example, increasing the concentration of nutrients and pollutants, and concentrating dolphins into deep pools that are also important areas for fishing, thereby increasing the chances of negative human interactions [39]. The altered hydrological regime on the Indus River has likely reduced the complexity of hydrologic and geomorphologic habitat and ultimately also diminished its carrying capacity and ability to support large numbers of aquatic megafauna. To preserve aquatic biodiversity, river management is needed that focuses on restoring both the timing and duration of flood pulses, as well as on maintaining critical minimum flows in the dry season.

The persistence of dolphins in the Beas River, India is likely to be due to the presence of constant water supplies little depleted by diversions. Dolphins in the Beas River occur in an isolated habitat fragment as the river downstream is virtually dry, and only connected with the rest of the river system for a few weeks each year during the monsoon floods. This demonstrates that in the presence of sufficient water, and an absence of other threats, river dolphins can persist for decades even in relatively small fragments of habitat near the periphery of their range. This subpopulation is of conservation importance, as all other Indus dolphins occur in a single river, the Indus, and are therefore at risk from environmentally correlated catastrophic events [40]. However, based on the historical pattern of decline, Indus dolphins are most likely to disappear in the future from locations with low river discharge located closer to the range periphery, meaning that dolphins in the Beas (close to range periphery with moderate discharge) and between Sukkur and Kotri Barrages (with low discharge located a moderate distance from the former range edge) are most at risk.

The date of habitat fragmentation was not selected by any of the models as a strong predictor of whether dolphins are still present. However depleted river discharge and habitat fragmentation by barrages are inextricably intertwined as barrages are responsible for diverting water, and they are a physical barrier that greatly impedes or prevents the dispersal of dolphins out of impacted river reaches.

### Population extirpation, core habitat and conservation

Contraction of geographic range is one of the principal characteristics exhibited by declining or threatened species [41], [42]. In general, at the periphery of a species geographic range, populations occupy less favourable habitat and occur at lower and more variable densities. Therefore, as a species becomes endangered it is expected that its geographic range will contract inwards, and that populations will persist in the range core until the final stages of decline [43]. However, for many endangered mammals the pattern of range decline is instead dictated by the spread of factors driving the decline, with those populations last impacted, regardless of their location, persisting longer than those that were historically large [41], [43]. The range of the Indus dolphin has also contracted inwards, and dolphins persist primarily in what is assumed to be the former range core or higher density area, however this is likely to be because the greatest threat, water extraction, is concentrated in the periphery of the subspecies range. This conclusion is supported by the continued persistence of the Beas River population at the range limit. However, that animals naturally occur at lower density in upstream areas and smaller rivers and are therefore more vulnerable near the range limit is also certainly a factor. The spatial pattern of Indus River dolphin decline is very different from the gradual decline in abundance described for the Yangtze River dolphin [16].

One of the greatest challenges in conservation science involves disentangling the relative contributions of multiple factors in the decline of species, especially when causes interact or vary spatially and temporally with importance [44]. Nevertheless, the primary factor identified in these models (i.e. low dry season discharge due to water diversion at barrages) is well supported, and is also the most salient for informing current ecosystem management. In the Mekong River, numerous stressors such as fisheries bycatch, hunting and habitat destruction have reduced the resident Irrawaddy dolphin (*Orcaella brevirostris)* population to less than 100 individuals [45]. The results of our study suggests that habitat fragmentation and/or flow disruption associated with the many proposed new dams on the Mekong are likely to further drive Irrawaddy dolphin decline potentially leading to local extinction. The Indus dolphin range decline is probably most prescient for the related Ganges dolphin (*Platanista gangetica gangetica*) which occurs in the neighbouring Brahmaputra and Ganges River systems and is subject to the same threats. The Ganges River is fragmented by barrages and flow is severely reduced in many areas, and the Brahmaputra River system is the focus of massive hydropower development [46], [47]. The range of the Ganges dolphin has begun to decline especially at the upper limit of the distributional range [48], and if water development continues as planned the range of the dolphin is expected to continue to shrink towards larger habitat fragments with higher discharge that are primarily in downstream locations. The results of this study suggest that other vertebrate populations in other large rivers, such as the Amazon, Orinoco and Ayeyarwady, will also respond with dramatic declines in range when dams and other water developments that reduce discharge, fragment habitat and change the hydrological regime are constructed.

The amount of habitat fragmentation and level of water withdrawals from rivers in Pakistan is extreme, negatively affecting human communities, eroding the delta, destroying fisheries and concentrating pollutants. This study indicates that if water development plans in South Asia and the wider Himalayan region proceed as currently proposed [10], [13]and follow the pattern demonstrated by the Indus, the resulting habitat fragmentation and flow disruption will likely cause large declines in resident freshwater cetaceans and other freshwater dependent species. Healthy rivers are of great importance to communities, and it is critical that where developments are planned, environmental flow and impact assessments be conducted that balance human requirements for irrigation water and power with the habitat requirements of the aquatic ecosystem that are vital to humans.

## Supporting Information

Table S1 **Details of extant and extirpated Indus dolphin subpopulations.**
(DOCX)

## References

[1] Sala OE, Chapin FS, Armesto JJ, Berlow R, Bloomfield J, et al.. (2000) Global biodiversity scenarios for the year 2100. Science 287. 10.1126/science.287.5459.1770 10710299

[2] Dudgeon D, Arthington AH, Gessner MO, Kawabata Z-I, Knowler DJ, et al (2006) Freshwater biodiversity: importance, threats, status and conservation challenges. Biological Reviews 81: 163–182. 16336747 10.1017/S1464793105006950

[3] Tilman D, May RM, Lehman CL, Nowak MA (1994) Habitat destruction and the extinction debt. Nature 371: 65–66.

[4] Strayer DL, Dudgeon D (2010) Freshwater biodiversity conservation: recent progress and future challenges. Journal of the North American Benthological Society 29: 344–358.

[5] Gibson L, Lynam AJ, Bradshaw CJA, He F, Bickford DP, et al (2013) Near-complete extinction of native small mammal fauna 25 years after forest fragmentation. Science 341: 1508–1510. 24072921 10.1126/science.1240495

[6] Prugh LR, Hodges KE, Sinclair ARE, Brashares JS (2008) Effect of habitat area and isolation on fragmented animal populations. PNAS 105: 20770–20775. 19073931 10.1073/pnas.0806080105 PMC2634894

[7] FAO (2012) Coping with water scarcity. An action framework for agriculture and food security. Rome, Italy: FAO Water Reports No.38.

[8] World Water Assessment Programme (2012) The United Nations World Water Development Report 4. Managing water under uncertainty and risk. Paris: UNESCO.

[9] Dudgeon D (2000) Large-scale hydrological changes in tropical Asia: prospects for riverine biodiversity. Bioscience 50: 793–806.

[10] Dutta AP (2010) Reservoir of Dams. Down to Earth May 1–15: 32–39.

[11] Verma S, Kampman DA, van der Zaag P, Hoekstra AY (2009) Going against the flow: A critical analysis of inter-state virtual water trade in the context of India's National River Linking Program. Physics and Chemistry of the Earth 34: 261–269.

[12] Dharmadhikary S (2008) Mountains of Concrete: Dam building in the Himalayas. Berkeley, CA: International Rivers. 48 p.

[13] Ziv G, Baran E, Nam S, Rodríguez-Iturbe I, Levin SA (2012) Trading-off fish biodiversity, food security, and hydropower in the Mekong River basin. PNAS www.pnas.org/cgi/doi/10.1073/pnas.1201423109. 10.1073/pnas.1201423109 PMC3326487 22393001

[14] IUCN (2013) IUCN Red List of Threatened Species. version 2013.2 <http://www.iucnredlist.org> Downloaded on 6 January 2014.

[15] Turvey ST, Pitman RL, Taylor BL, Barlow J, Akamatsu T, et al (2007) First human-caused extinction of a cetacean species? Biology Letters 3: 537–540. 17686754 10.1098/rsbl.2007.0292 PMC2391192

[16] Turvey ST, Barrett LA, Hart T, Collen B, Yujiang H, et al (2010) Spatial and temporal extinction dynamics in a freshwater cetacean. Proceedings of the Royal Society B 277: 3139–3147. 20484234 10.1098/rspb.2010.0584 PMC2982057

[17] Anderson J (1879) Anatomical and zoological researches: comprising an account of the zoological results of the two expeditions to western Yunnan in 1868 and 1875 and a monograph of the two cetacean genera *Platanista* and *Orcella*. Bernard Quaritch, Piccadilly, London.

[18] Reeves RR, Chaudhry AA, Khalid U (1991) Competing for water on the Indus Plain: Is there a future for Pakistan's river dolphins? Environmental Conservation 18: 341–349.

[19] Braulik GT (2006) Status assessment of the Indus River dolphin, *Platanista gangetica minor*, March-April 2001. Biological Conservation 129: 579–590.

[20] Behera SK, Nawab A, Rajkumar B (2008) Preliminary investigations confirming the occurrence of Indus River dolphin *Platanista gangetica minor* in River Beas, Punjab, India. Journal of the Bombay Natural History Society 105: 90–126.

[21] Reeves RR, Leatherwood S (1994) Dams and river dolphins: can they co-exist? Ambio 23: 172–175.

[22] Braulik GT, Bhatti ZI, Ehsan T, Hussain B, Khan AR, et al (2012) Robust abundance estimate for endangered river dolphin subspecies in South Asia. Endangered Species Research 17: 201–215.

[23] Collen B, Purvis A, Mace GM (2010) When is a species really extinct? Testing extinction inference from a sighting record to inform conservation assessment. Diversity and Distributions 16: 755–764.

[24] Butchart SHM, Stattersfield AJ, Brooks TM (2006) Going or gone: defining 'Possibly Extinct' species to give a truer picture of recent extinctions. Bulletin-British Ornithologists Club 126: 7–24.

[25] IUCN (2011) Pakistan Water Gateway: Gateway to water information about Pakistan. www.waterinfo.net.pk.

[26] Braulik GT, Reichert AP, Ehsan T, Khan S, Northridge SP, et al (2012) Habitat use by a freshwater dolphin in the low-water season. Aquatic Conservation: Marine and Freshwater Ecosystems 22: 533–546.

[27] Crawley MJ (2007) The R Book. Chichester, England: John Wiley & Sons Ltd. 951 p.

[28] R Development Core Team (2012) R: A language and environment for statistical computing. Vienna, Austria: R Foundation for Statistical Computing.

[29] Burnham KP, Anderson DR (2002) Model selection and multi- model inference: a practical information-theoretic approach. 2^nd^ edition. New York: Springer-Verlag. 496 p.

[30] Fox J (2008) Applied regression analysis and Generalised Linear Models. 2nd edition. Los Angeles: Sage Publications, Inc. 665 p.

[31] Directorate of Land Reclamation Punjab (2007) Surface water quality monitoring plan. Lahore: Irrigation and Power Department, Government of Punjab. 27 p.

[32] Read AJ (2008) The looming crisis: interactions between marine mammals and fisheries. Journal of Mammalogy 89: 541–548.

[33] Khan H (1947) A fishery survey of River Indus. Journal of the Bombay Natural History Society 46: 529–535.

[34] Pilleri G (1972) Field observations carried out on the Indus dolphin *Platanista indi* in the Winter of 1972. Investigations on Cetacea 4: 23–29.

[35] Braulik GT (2012) Conservation ecology and phylogenetics of the Indus River dolphin (*Platanista gangetica minor*). St. Andrews, UK. 267: University of St. Andrews.

[36] Reeves RR (1991) Conservation of the bhulan (blind river dolphin) in the Punjab. Natura. 3–22.

[37] Nilsson C, Reidy CA, Dynesius M, Revenga C (2005) Fragmentation and flow regulation of the world's large river systems. Science 308: 405–408. 15831757 10.1126/science.1107887

[38] Xenopoulos MA, Lodge DM (2006) Going with the flow: using species-discharge relationships to forecast losses in fish biodiversity. Ecology 87: 1907–1914. 16937627 10.1890/0012-9658(2006)87[1907:gwtfus]2.0.co;2

[39] Kelkar N, Krishnaswamy J, Choudhary S, Sutaria D (2010) Coexistence of fisheries with river dolphin conservation. Conservation Biology 24: 1130–1140. 20337677 10.1111/j.1523-1739.2010.01467.x

[40] Gilpin ME (1990) Extinction in finite metapopulations living in correlated environments. In: Shorrocks B, Swingland IR, editors. Living in a patchy environment. New York: Oxford University Press. 177–187.

[41] Channell R, Lomolino MV (2000) Trajectories to extinction: spatial dynamics of the contraction of geographical ranges. Journal of Biogeography 27: 169–179.

[42] Simberloff D (1986) The proximate causes of extinction. In: Raup DM, Jablonski D, editors. Patterns and processes in the history of life. Berlin: Springer-Verlag. 259–276.

[43] Lomolino MV, Channell R (1995) Splendid isolation: patterns of geographic range collapse in endangered mammals. Journal of Mammalogy 76: 335–347.

[44] Johnson PTJ, McKenzie VJ, Peterson AC, Kerby JL, Brown J, et al (2010) Regional decline of an iconic amphibian associated with elevation, land-use change and invasive species. Conservation Biology 25: 556–566. 10.1111/j.1523-1739.2010.01645.x 21342266

[45] Ryan GE, Dove V, Trujillo F, Doherty Jr PF (2011) Irrawaddy dolphin demography in the Mekong River: an application of mark-resight models. Ecosphere 2: 1–15.

[46] Bashir T (2010) Ganges river dolphin (Platanista gangetica) seeks help. Current Science 98: 287–288.

[47] Baruah D, Hazarika LP, Bakalial B, Borah S, Dutta R, et al (2012) A grave danger for the Ganges dolphin (Platanista gangetica, Roxburgh) in the Subansiri River due to a large hydroelectric project. Environmentalist 32: 85–90.

[48] Sinha RK, Smith BD, Sharma G, Prasad K, Choudhury BC, et al. (2000) Status and distribution of the Ganges Susu (*Platanista gangetica*) in the Ganges River system of India and Nepal. In: Reeves RR, Smith BD, Kasuya T, editors. Biology and Conservation of Freshwater Cetaceans in Asia. Gland, Switzerland & Cambridge, U.K.: IUCN. 54–61.

