# Supplementary material for: Habitat Fragmentation and Species Extirpation in Freshwater Ecosystems; Causes of Range Decline of the Indus River Dolphin (Platanista gangetica minor)
Source: PLoS One. 2014 Jul 16;9(7):e101657. doi: 10.1371/journal.pone.0101657 (PMC4100755; doi:10.1371/journal.pone.0101657)
Supplement: Table S1 — Details of extant and extirpated Indus dolphin subpopulations. (DOCX) [file pone.0101657.s001.docx]

Table S1 – Details of extant and extirpated Indus dolphin subpopulations

| **#** | **Subpopulation** | **Date ^a^ Isolated** | **Extant (1) Extirpated (0)** | **Last Sighting Date^b^** | **Time to ^c^ Extirpation** | **Length (km)^d^** | **River Size^e^** | **Conf^f^** | **Median ^g^ Discharge** | **Dist**  **(km)^h^** | **Slope**  **m/km** |
| --- | --- | --- | --- | --- | --- | --- | --- | --- | --- | --- | --- |
| **13** | Balloki-Sidhnai | 1886 | 0 | 1962 | 76 | 175 | 7 | 0 | 0 | 175 | 0.29 |
| **11** | Marala-Khanki | 1892 | 0 | 1879 | N/K | 35 | 26 | 0 | 4391 | 0 | 0.54 |
| **8** | Upstream Rasul | 1901 | 0 | 1975 | 74 | 50 | 23 | 0 | 27000 | 71 | 0.72 |
| **15** | Hussainiwala-Suleimanki | 1926 | 0 | 1988 | 62 | 110 | 14 | 0 | 0^k^ | 287 | 0.18 |
| **14** | Suleimanki-Islam | 1927 | 0 | 1972 | 45 | 145 | 14 | 0 | 0 | 438 | 0.26 |
| **17** | Ropar,ShahNehar-Harike | 1927^i^ | 1 | N/A | N/A | 220 | 14 | 1 | 15414^k^ | 148 | 0.38 |
| **10** | Islam,Sidhnai,Trimmu-Panjnad | 1933 | 0 | 1981 | 48 | 435 | 26 | 2 | 2259 | 610 | 0.11 |
| **9** | Rasul,Qadirabad-Trimmu | 1939 | 0 | 1975 | 36 | 490 | 26 | 1 | 7990 | 327 | 0.13 |
| **1** | Upstream Jinnah | 1946 | 0 | 1955 | 9 | 35 | 93 | 0 | 38000 | 35 | 0.89 |
| **16** | Harike-Hussainiwala | 1955 | N/K | N/K | N/K | 30 | 14 | 0 | 0^k^ | 184 | 0.33 |
| **6** | Sukkur-Kotri | 1955 | 1 | N/A | N/A | 318 | 93 | 0 | 7224 | 520 | 0.09 |
| **7** | Downstream Kotri | 1955 | 0 | N/K | N/K | 222 | 93 | 0 | 0 | 205 | 0.09 |
| **3** | Chashma-Taunsa | 1959 | 1 | N/A | N/A | 230 | 93 | 0 | 43000 | 351 | 0.23 |
| **4** | Taunsa-Guddu | 1962 | 1 | N/A | N/A | 277 | 93 | 1 | 38093 | 636 | 0.21 |
| **5** | Guddu-Sukkur | 1962 | 1 | N/A | N/A | 126 | 93 | 0 | 34214 | 645 | 0.17 |
| **12** | Khanki-Qadirabad | 1967 | 0 | 1879 | N/K | 45 | 26 | 0 | 578 | 0 | 0.44 |
| **2** | Jinnah-Chashma | 1971 | 1 | N/A | N/A | 60 | 93 | 0 | 47039 | 122 | 0.45 |

# Dolphin subpopulation number, shown in Fig 1, ^a^ Date that dolphins were confined between two barrages, taken as the completion date of the downstream barrage, ^b^ Most recent dolphin sighting, where sighting dates were imprecise they were rounded, for example to 1972, 1975 or 1978. ^C^ The time that elapsed from the isolation date to the last sighting date ^d^ Length of river between barrages ^e^ Annual average flows calculated by averaging daily flows for the period 1922 to 1961 (IUCN 2011). ^f^ Number of river confluences occurring within river section ^g^ Median daily discharge between October 1^st^ and March 31^st^ (the dry season) from the upstream barrage in cubic feet per second.  ^h^Distance to the former range limit described by Anderson (1879)(Fig. 1). ^i^ Harike isolation date taken from completion date of Hussainiwala barrage nearby. ^k^ Median monthly discharge between October 1^st^ and March 31^st^ in cubic feet per second. N/K = not known. N/A = not applicable

Table A2 – Current and former fragments of Indus dolphin habitat listed in chronological order of their creation. River sections highlighted grey are still present

| **#** | **Fragment Description** | **Creation Date** | **End Date** | **Duration** | **Length** | **Dolphin extant** |
| --- | --- | --- | --- | --- | --- | --- |
| **0** | Former un-fragmented range | N/A | 1886 | N/A | 3208 | 1 |
| **1** | Sidhnai to Madhopur | 1886 | 1917 | 31 | 380 | 1 |
| **2** | Former range #0, minus #1 | 1886 | 1892 | 6 | 2828 | 1 |
| **3** | Khanki to Marala | 1892 | 2011 | 119 | 35 | 0 |
| **4** | Former range #0, minus #1 & 3 | 1892 | 1901 | 9 | 2793 | 1 |
| **5** | Upstream Rasul | 1901 | 2011 | 110 | 50 | 0 |
| **6** | Former range #0, minus #1, 3 & 5 | 1901 | 1926 | 25 | 2743 | 1 |
| **7** | Sidhnai to Balloki | 1917 | 2011 | 94 | 175 | 0 |
| **8** | Upstream Suleimanki | 1926 | 1927 | 1 | 360 | 1 |
| **9** | Former range, minus #3, 5, 7 & 8 | 1926 | 1927 | 1 | 2383 | 1 |
| **10** | Suleimanki to Hussainiwala | 1927 | 2011 | 84 | 110 | 0 |
| **11** | Upstream Hussainiwala | 1927 | 1955 | 28 | 250 | 1 |
| **12** | Islam to Suleimanki | 1927 | 2011 | 84 | 145 | 0 |
| **13** | Former range, minus everything upstream of Islam, Sidhnai, Rasul & Khanki barrages | 1927 | 1932 | 5 | 2238 | 1 |
| **14** | Downstream Sukkur to sea | 1932 | 1955 | 23 | 540 | 1 |
| **15** | Former range, minus everything upstream Islam, Sidhnai, Rasul & Khanki barrages, and downstream Sukkur | 1932 | 1933 | 1 | 1698 | 1 |
| **16** | Panjnad to Islam/Sidhnai/Rasul/Khani | 1933 | 1939 | 6 | 970 | 1 |
| **17** | All Indus River to Sukkur & Panjnad | 1933 | 1946 | 13 | 728 | 1 |
| **18** | Panjnad to Trimmu/Sidhnai/Islam | 1939 | 2011 | 72 | 435 | 0 |
| **19** | Trimmu to Rasul/Khanki | 1939 | 1967 | 28 | 535 | 1 |
| **20** | Upstream Jinnah | 1946 | 2011 | 65 | 35 | 0 |
| **21** | Jinnah to Sukkur & Panjnad | 1946 | 1959 | 13 | 693 | 1 |
| **22** | Sukkur to Kotri | 1955 | 2011 | 56 | 318 | 1 |
| **23** | Downstream Kotri to sea | 1955 | 2011 | 56 | 222 | 0 |
| **24** | Hussainiwala to Harike | 1955 | 2011 | 56 | 30 | 0 |
| **25** | Upstream Harike | 1955 | 2011 | 56 | 220 | 1 |
| **26** | Taunsa to Sukkur & Panjnad | 1959 | 1962 | 3 | 403 | 1 |
| **27** | Jinnah to Taunsa | 1959 | 1971 | 12 | 290 | 1 |
| **28** | Guddu to Sukkur | 1962 | 2011 | 49 | 126 | 1 |
| **29** | Taunsa to Guddu & Panjnad | 1962 | 2011 | 49 | 277 | 1 |
| **30** | Trimmu to Rasul & Qadirabad | 1967 | 2011 | 44 | 490 | 0 |
| **31** | Qadirabad to Khanki | 1967 | 2011 | 44 | 45 | 0 |
| **32** | Taunsa to Chashma | 1971 | 2011 | 40 | 230 | 1 |
| **33** | Jinnah to Chashma | 1971 | 2011 | 40 | 60 | 1 |

Note: Lengths listed here were measured using ArcView 3.2 and satellite images, and are shorter than those recorded during vessel-based surveys in the same sections of river. N/A = Not applicable.
